# On the Activation Energy of Termination in Radical Polymerization, as Studied at Low Conversion

**DOI:** 10.3390/polym16223225

**Published:** 2024-11-20

**Authors:** Majed M. Alghamdi, Gregory T. Russell

**Affiliations:** 1 Department of Chemistry, Faculty of Science, King Khalid University, P.O. Box 9004, Abha 61413, Saudi Arabia; mmalghamdi@kku.edu.sa; 2 School of Physical and Chemical Sciences, University of Canterbury, Private Bag 4800, Christchurch 8140, New Zealand

**Keywords:** radical polymerization, termination rate coefficient, chain-length dependence, activation energy

## Abstract

The chain-length-dependent nature of the termination reaction in radical polymerization (RP) renders the overall termination rate coefficient, <*k*_t_>, a complex parameter in the usual situation where the radical chain-length distribution is non-uniform. This applies also for the activation energy of termination, *E*_a_(<*k*_t_>), which we subject to detailed mechanistic investigation for the first time. The experimental side of this work measures *E*_a_(<*k*_t_>) for the dilute-solution, low-conversion, chemically initiated homopolymerization of styrene (ST), methyl methacrylate (MMA), butyl methacrylate, and dodecyl methacrylate. Values of 25–39 kJ mol^−1^ are obtained, consistent with strong chain-length-dependent termination (CLDT) for short chains. On other hand, the reanalysis of analogous bulk polymerization data for ST and MMA finds *E*_a_(<*k*_t_>) values of 18–24 kJ mol^−1^, consistent with weak CLDT for long chains. Both these results are as expected from the so-called composite model for CLDT. A simple analytic framework for understanding and predicting *E*_a_(<*k*_t_>) values is presented for the standard RP situation of continuous initiation. All the results of this work can be rationalized via this framework, which clearly establishes that *E*_a_(<*k*_t_>) is determined by far more than just the *E*_a_ of radical diffusion. This framework is extended to activation energy for the number-average degree of polymerization, *E*_a_(*DP*_n_), which we measure and successfully scrutinize via our CLDT model. In the final section of this work, we make interesting, testable predictions about *E*_a_(<*k*_t_>) and/or *E*_a_(*DP*_n_) in various RP systems of different natures to those studied here, most notably, systems involving acrylates, continuous photoinitiation, or dominant chain transfer.

## 1. Introduction

As it is used for approximately half of the total synthetic polymer production, the high importance of radical polymerization (RP) is very well known. Accordingly, the study of RP kinetics has been a topic of keen interest for three quarters of a century [1]. Common knowledge is that the three fundamental reactions in this process are initiation, propagation, and termination. Given how pivotal this process is, it is astounding that fundamental aspects of it are still not grasped. For example, what is understood about the activation energy of termination, the fundamental reaction in which two propagating radicals react together so that the activity of each is lost and thus cease growing? Being a diffusion-controlled reaction, most workers would just say that the activation energy must be that of the relevant diffusion process, and indeed this approach is commonly used in modelling [2]. However, that it cannot be this simple is immediately clear from the well-known results [3,4].

(1) $$\langle k_{t}\rangle =\sum_{i=1}^{\infty }\sum_{j=1}^{\infty }k_{t}^{i,j}\frac{c_{R^{i}}}{c_{R}}\frac{c_{R^{j}}}{c_{R}}$$

This equation, which holds at all times, expresses the overall rate coefficient for termination, <*k*_t_>, in terms of the underlying microscopic termination rate coefficients, *k*_t_*^i,j^*, and radical concentrations, $c_{R^{i}}$, where the former denotes the rate coefficient for termination between a radical of chain length *i* and another of chain length *j*, the latter is the concentration of radicals of chain length *i*, and *c*_R_ is the total radical concentration. The origin of Equation (1) resides in the termination being chain-length-dependent in rate, something that has long been suspected [3] and, in recent decades, has been definitively proven experimentally, as summarized in recent reviews [4,5]. This work is concerned with the activation energy of <*k*_t_>, *E*_a_(<*k*_t_>), because <*k*_t_> is the quantity that generally is measured and deployed in process modelling.

Looking at Equation (1), it is evident that the variation of <*k*_t_> with temperature, *T*, cannot be straightforward. Most obviously, this is the case because multifarious *k*_t_*^i,j^* go into determining <*k*_t_>, and one cannot expect that all of them have the same *E*_a_, since the nature of the diffusion process that determines the value of *k*_t_*^i,j^* must change with the chain length. But, even if one assumes that all *k*_t_*^i,j^* have the same *E*_a_, there are still the terms $c_{R^{i}}$/*c*_R_ in Equation (1). This ratio is the fraction of polymerizing radicals of chain length i, i.e., the (living) radical chain-length distribution (RCLD). That there is extensive variation in this distribution with *T* is most obviously evidenced by the major variation in the number-average degree of polymerization, *DP*_n_, of dead polymer produced by RP. Any factor that alters the RCLD must alter <*k*_t_> and thus influence *E*_a_(<*k*_t_>). Such important factors include the initiator efficiency, *f*, rate coefficient for initiator decomposition, *k*_d_, rate coefficient for propagation, *k*_p_, and, of course, termination rate coefficients themselves. In this work, it is these subtle effects that will be the focus, and it will be seen that they have a compelling influence on *E*_a_(<*k*_t_>).

The diffusion-controlled nature of termination has led to the recognition of many factors that may influence <*k*_t_>, including viscosity, solvent interactions, chain flexibility, dynamics of entanglements, polymer weight fraction, and—of particular relevance—chain length [6]. However, the effect of the RCLD on <*k*_t_>, as captured in Equation (1), is relatively sparingly recognized, even though it is arguably responsible for a lot of the confounding complexities in RP kinetics [4,7]. Barely grasped at all is the effect of RCLD on the variation of <*k*_t_> with *T*, which is why we substantiate some earlier musings [8,9] in this work. We do this by measuring <*k*_t_> in continuously initiated polymerizations and by then seeking to understand the obtained *E*_a_ in terms of the latest understanding of chain-length-dependent termination (CLDT).

Because CLDT is central to this work, it is appropriate to summarize the state of play in this regard. Currently ascendant is the so-called composite model [10]:

(2a) $$k_{t}^{i,i}=k_{t}^{1,1}i^{-e_{S}},\;i\leq i_{c}$$

(2b) $$k_{t}^{i,i}=k_{t}^{1,1}{\left(i_{c}\right)}^{\left(-e_{S}+e_{L}\right)}i^{-e_{L}},\;i>i_{c}$$

This model posits that to achieve a good approximation, there are two distinct regimes in the variation in the homotermination rate coefficient, *k*_t_*^i,i^*, with *i*, namely a power-law variation with exponent *e*_S_ for short chains (Equation (2a)), while beyond a crossover chain length, *i*_c_, there is a different power-law variation, exponent *e*_L_, for long chains (Equation (2b)). Consistent with the meaning of *k*_t_*^i,i^*, the fourth parameter above, *k*_t_^1,1^, is the rate coefficient for termination between monomeric radicals, which have *i* = 1. In the event of *e*_S_ = *e*_L_, Equation (2) simplifies to one of the original models for CLDT [4] as follows:

(3) $$k_{t}^{i,i}=k_{t}^{1,1}i^{-e}$$

As will be seen, Equation (3) is still of importance, even if it has been superseded.

Subsequent to its proposal, Equation (2) has been extensively verified for a considerable number of monomers thanks to the development of advanced techniques such as single-pulse pulsed-laser polymerization combined with EPR spectroscopy (SP-PLP-EPR) [11,12,13,14,15], SP-PLP performed with a reversible addition-fragmentation chain-transfer agent combined with NIR spectroscopy (SP-PLP-NIR-RAFT) [16,17,18], and steady-state RAFT polymerization (RAFT-CLD-T) [19,20]. There have been several reviews of this work [4,5,21]. In particular, these techniques have been deployed for examining the monomers methyl methacrylate (MMA) [22,23,24] and styrene (ST) [25,26] at low conversion. For long chains, there is a wealth of data supporting *e*_L_ ≈ 0.16–0.20 [4,7,11,22,27,28]. For small chains in ST and methacrylate systems, it has been found that *e*_S_ ≈ 0.5–0.65, which also agrees with the theoretical expectations [4,10]. These values will be of importance in this work, as will the measured value *i*_c_ ≈ 50–200 [4].

The aforementioned techniques through which Equation (2) has found verification all involve the narrowing of the RCLD to such an extent that the observed <*k*_t_> at any instant is essentially a *k*_t_*^i,i^* value. However, the standard situation for producing polymer involves continuous initiation; for example, via thermally decomposing chemical initiator, which means that the RCLD is broad. How do the <*k*_t_> values obtained in such circumstances shed light on CDLT, i.e., on the underlying *k*_t_*^i,j^* values, and are such findings consistent with results from the niche techniques used to determine *k*_t_*^i,i^*? In this work, we investigate these questions via the measurement and analysis of *E*_a_(<*k*_t_>) values, as obtained from straightforward determinations of the rate of polymerization, *R*_p_, in thermally induced systems. As well as contributing to the building up of a comprehensive database of steady-state <*k*_t_> values, we aim to show that variations in *E*_a_(<*k*_t_>) can be explained in terms of CLDT concepts, thereby providing further insight into the mechanism of termination. We have a particular focus on systems with *DP*_n_ < *i*_c_, because the behaviour of such systems should be significantly influenced by Equation (2a) [10], for which *e* is higher than for long chains, as already explained. In our thermally induced polymerizations, we achieve *DP*_n_ < 100 ≈ *i*_c_ through employing relatively high *T* and through adding solvent to attain a low monomer concentration, *c*_M_.

## 2. Materials and Methods

### 2.1. Materials

2,2′-Azobisisobutyronitrile (AIBN) was purified via recrystallisation. Bis(3,5,5-trimethylhexanoyl) peroxide (BTMHP) (Akzo Nobel, Amersfoort, The Netherlands) was utilized as received. Styrene (ST; Sigma-Aldrich, Auckland, New Zealand), methyl methacrylate (MMA; Sigma-Aldrich), *n*-butyl methacrylate (BMA; Sigma-Aldrich), and dodecyl methacrylate (DMA; Sigma-Aldrich) were obtained as indicated. In order to remove the inhibitor, all these monomers were purified chromatographically. The solvents trifluorotoluene (TFT; Sigma-Aldrich, ≥99.0%) and ethylbenzene (EBz, Fluka, Buchs, Switzerland) were utilized as received.

### 2.2. Polymerizations

A series of radical homopolymerizations with different monomers were conducted isothermally in solution at atmospheric pressure, employing either AIBN or BTMHP as the initiator and either EBz (for ST) or TFT (all three methacyrlates) as the solvent. Details about these solvent choices will be presented in due course. In order to identify the impact of temperature on the termination rate coefficient, polymerization was studied at temperatures ranging from 50 to 90 °C. Prior to use, reaction mixtures were thoroughly deoxygenated by purging with nitrogen gas for 15 min. The reaction mixtures were then divided into several samples, typically 5–10. The polymerization was carried out for each sample and stopped at a different time by immersing the sample in ice water. The stoppage times were chosen so as to maintain dilute-solution conditions; specifically, the conversion of the monomer into a polymer was kept below 20% (which, due to the high level of solvent, still results in a dilute solution). A negligible change in volume upon heating to the reaction temperature was assumed for all polymerizations, meaning that *c*_M_ and initiator concentration, *c*_I_, were taken as being unchanged from the preparation conditions. The conversion for each sample was determined using gravimetric analysis, after evaporating the residual monomer and solvent and then drying the sample in a vacuum oven.

### 2.3. Molar Mass Measurement

Size exclusion chromatography (SEC) analysis was conducted on selected samples. The instrument was equipped with a refractive index (RI) detector, and the system included 2 × Polypore 300 × 7.5 mm columns with a nominal particle size of 5 μm. Tetrahydrofuran (THF) was used as the eluent at a flow rate of 1.0 mL min^−1^, and the analysis was conducted at 35 °C with a polymer concentration of approximately 5 mg mL^−1^. Calibration was via polystyrene standards; for poly(MMA), poly(BMA), and poly(DMA) samples, universal calibration was employed with Mark–Houwink parameters, as in Table S1.

Electrospray ionization mass spectrometry (ESI-MS) was conducted as detailed in the Supporting Information.

## 3. Data Analysis and Results

The following form of the rate law for steady-state RP was utilized for analyzing conversion-time data [29]:

(4) $$\frac{-dln(1-x)}{dt}=k_{p}c_{R}=k_{p}{\left(\frac{fk_{d}c_{I}}{\langle k_{t}\rangle }\right)}^{0.5}\equiv k_{o}$$

**Figure 1:**
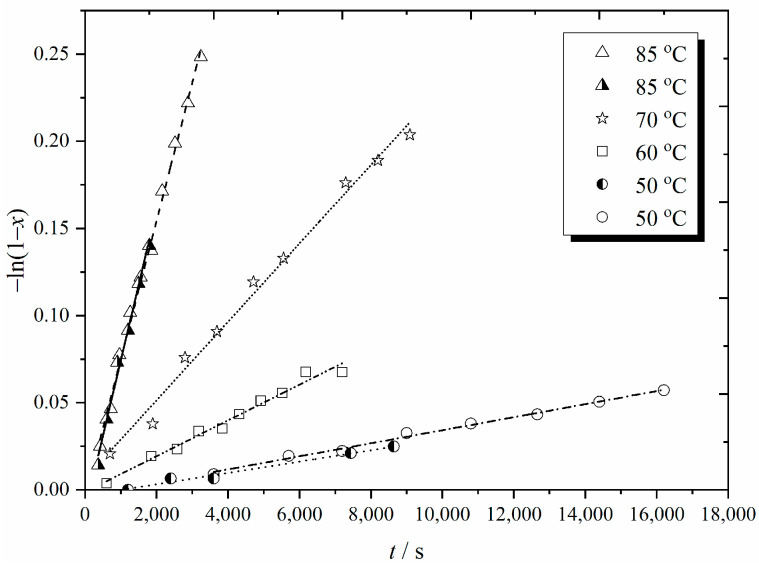
Fractional conversion (*x*)-time (*t*) data from the radical polymerization of styrene (ST) at various temperatures as indicated, where *c*_AIBN_ = 0.05 mol L^−1^ and *c*_ST_ = 0.67 mol L^−1^ with ethyl benzene (EBz) as the solvent were used. Points: experimental measurements; lines: best fit for each set of results. Note that the two lines and sets of symbols at 50 and 85 °C represent duplicate runs.

Here, *x* represents the fractional conversion of a monomer into a polymer, *t* denotes time, *c*_R_ is the overall radical concentration, and <*k*_t_>, *k*_p_, and *k*_d_ are the rate coefficients for termination, propagation, and initiator decomposition, respectively, while the initiator efficiency and concentration are *f* and *c*_I_, respectively. Thus, *x*(*t*) data should be plotted as −ln(1 − *x*) versus *t*, a straight line fitted, and *k*_o_ for the polymerization obtained as the slope. Typical results are presented in Figure 1, in which it is evident that the expectation of linear behaviour is met. Furthermore, the well-known rate increase with temperature is immediately visible, and there is good experimental reproducibility (see the data at 50 and 85 °C). This gives confidence in the conclusions drawn from such experiments, and, once again, indicates that when carried out attentively, gravimetry is no less precise than many other methods for monitoring conversion and studying kinetics [9,29].

Results from every low-conversion solution polymerization carried out in this work are presented in Table 1. From each *k*_o_, an experimentally measured value of <*k*_t_> may be obtained using the following rearranged form of Equation (4):

(5) $$\langle k_{t}\rangle =fk_{d}c_{I}{\left(\frac{k_{p}}{k_{o}}\right)}^{2}$$

Using this equation requires knowledge of *f*, *k*_d_, and *k*_p_, which, in our case, are needed as a function of temperature and for a variety of monomers and initiators. These variations were stipulated via Arrhenius expressions:

(6) $$k=Aexp\left(\frac{-E_{a}}{RT}\right)$$

The employed values of *A* and *E*_a_ for all RP rate parameters for all systems studied here are given in Table 2, and the resulting <*k*_t_> values are given in Table 1.

The values for *k*_p_ and *k*_d_ in Table 2 are well established, although further will be said about *k*_d_ (AIBN) below. However, *f* is rarely measured properly, and, consequently, Arrhenius parameters for it are virtually unreported. For AIBN, we fitted experimentally obtained *f*(*T*) in bulk styrene [34] to Equation (6), which resulted in the Arrhenius parameters of Table 2. This expression generates *f* = 0.80 at 100 °C, which agrees well with another study [38]. However, our fit should not be used above 100 °C, because it will soon generate *f* > 1, which is physically impossible. It is stressed that these *f* values are for low conversion only, because *f* decreases with conversion [34]. It also decreases with an increasing viscosity [34], but, in fact, all the systems of this work have very similar viscosities because the monomers are diluted in either TFT (0.57 cP at 20 °C) or EBz (0.67 cP at 20 °C), both of which are similar to styrene (0.76 cP at 20 °C), in which the measurements of *f* were made [34]. Thus, these values should hold reasonably accurately for all our systems without adjustment. For BTMHP, there is an absence of information about the effect of temperature on *f*, so consequently, the reported value *f* = 0.53 [35] was utilized at all temperatures, resulting in the parameters of Table 2. Of course, *E*_a_(*f*) = 0 is not realistic, but the value is likely to be very small and of the magnitude of the AIBN value of 5.7 kJ mol^−1^, which means that the variation with temperature is minor in scale. Furthermore, it will be seen below that the <*k*_t_> values obtained with the two different initiators are in good agreement, which justifies *E*_a_(*f*) = 0 for BTMHP as a workable approximation.

It is noted that the reporting of *k*_o_—which is essentially the raw experimental output—in Table 1 will allow for the simple reprocessing of data if more accurate parameter values for use in Equation (5) become available in the future. Indeed, an example of this in relation to our previous work [9] will be provided below.

The other values in Table 1 are *DP*_n_. These were calculated using the well-known Mayo equation [39]:

(7) $$\frac{1}{{DP}_{n}}=C_{trM}+C_{trS}\frac{c_{S}}{c_{M}}+\frac{\left(1+\lambda \right){\left(fk_{d}c_{I}\langle k_{t}\rangle \right)}^{0.5}}{k_{p}c_{M}}$$

This equation assumes only steady-state and long chains, so it will be reasonably accurate for our work as long as the input parameter values are accurate, which we made every effort to achieve. Thus, we used *f*, *k*_d_, and *k*_p_ as already detailed and <*k*_t_> as measured. For the fraction of termination by disproportionation, *λ*, we used the accurately measured value 0.63 for MMA [35]. In the absence of any other good information, we made the reasonable assumption that this value also holds for BMA and DMA, as all these monomers are chemically similar. For ST, we used *λ* = 0.1 [40], consistent with it predominantly terminating by combination. Although it is expected that *λ* changes with temperature [40], there is no good information in the literature on this, and, furthermore, the variation will be small in absolute terms because just like *f*, the values are constrained to be between 0 and 1. Thus, we utilized the stated values for all temperatures.

Transfer processes are incorporated into Equation (7) via the so-called transfer constants, with *C*_trM_ for monomers and *C*_trS_ for solvents, at a certain concentration *c*_S_. Although these will not be zero, they can be assumed to be negligible for our conditions of low *c*_M_ and high *T*, which will result in relatively small *DP*_n_ and the so-called termination-control of dead-chain formation [7]. Although our high *c*_S_ potentially brings the transfer to solvent into play, we note that this is suppressed by the widespread use of TFT, which lacks labile C–H bonds for transfer [41]. Thus, we used *C*_trM_ = *C*_trS_ = 0 for calculations with Equation (7), and we note that if any significant dead-chain formation by transfer actually did occur, it would result in an even lower *DP*_n_ than those reported in Table 1.

Even if the *DP*_n_ are indicative calculations rather than precise measurements, it is evident that for the most part, we succeeded in our aim of establishing experimental conditions such that *DP*_n_ was of order *i*_c_ or lower, so that we could investigate the variation in <*k*_t_> with *T*, where *e*_S_—see Equation (2a)—plays a significant role in shaping the narrative.

**Figure 2:**
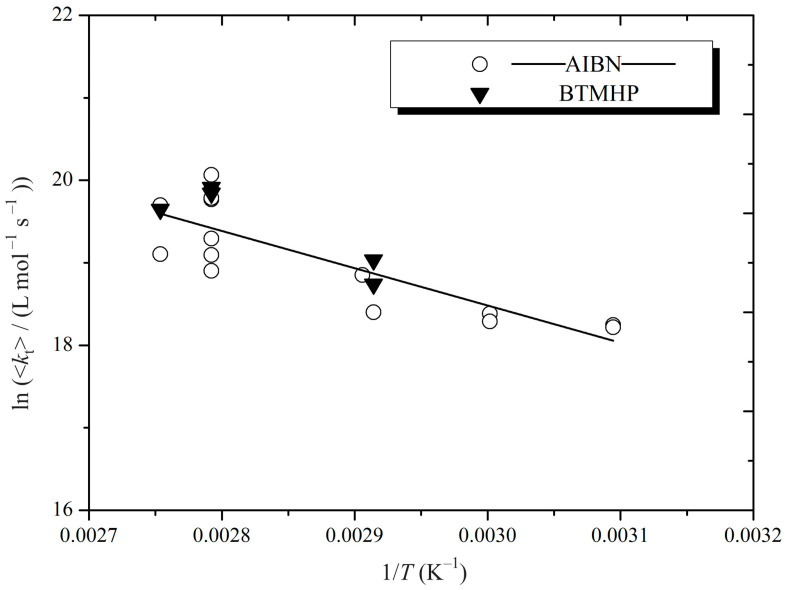
Arrhenius plot for the variation in the overall termination rate coefficient, <*k*_t_>, with temperature, *T*, for MMA polymerizations initiated by AIBN or BTMHP, as indicated (see Table 1 for further details on polymerization conditions).

Before proceeding, we present in Figure 2 all our <*k*_t_> results for MMA. The scatter in the data is actually very low by historical standards for <*k*_t_> [6,9]. Furthermore, the agreement between the two sets of results using different initiators, viz. AIBN and BTMHP, is excellent, where it should be noted that the different concentrations of each (0.05 and 0.018 mol L^−1^, respectively) were chosen so as to give very similar rates of initiation at each *T*, thereby eliminating any potential difference in <*k*_t_> due to this effect of CLDT [7]. The data of Figure 2 can therefore be said to evidence once again the reproducibility of our experimental results. Furthermore, these data generate confidence in the parameter values in Table 2 tha, which have been used to derive <*k*_t_> values from our *x*(*t*) measurements.

### Reanalysis of Previous Results

**Figure 3:**
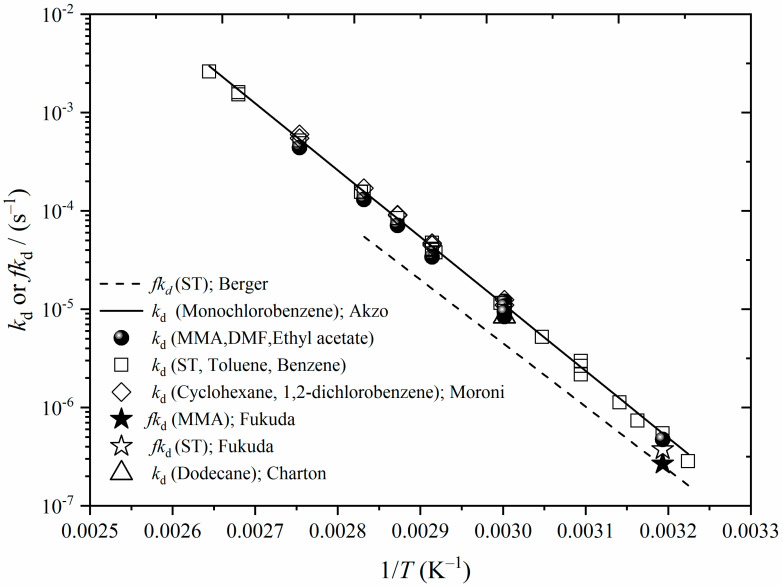
Arrhenius plot for variation in the initiator decomposition rate coefficient, *k*_d_, with temperature, *T*, for AIBN in various solvents by various workers as follows (from top of legend to bottom): in bulk styrene, by Berger [42] (presented as *fk*_d_ from [8], where *f* is initiator efficiency); chlorobenzene, AkzoNobel [33]; MMA and DMF, Szafko and Feist [49]; in MMA and in DMF, Moroni [48]; ethyl acetate, Bawn and Mellish [43]; ST, Breitenbach and Schindler [52]; ST/toluene, Moad et al. [47]; Tâlat-Erben and Bywater, toluene [50]; in toluene and in benzene, Van Hook and Tobolsky [51]; benzene, Bawn et al. [43,44]; benzene, Krstina et al. [46]; in cyclohexane and in dichlorobenzene, Moroni [48]; *fk*_d_ in MMA and in ST, Fukuda et al. [53]; and dodecane, Charton et al. [45].

Equation (5) makes clear that <*k*_t_> ~ *fk*_d_ in the processing of *k*_o_ values. Thus, any aberrant trend in *fk*_d_ values that are used in such processing will be carried over into the resulting <*k*_t_>. Since carrying out our earlier work on the variation in MMA and ST <*k*_t_> with *c*_I_, *c*_M_, and *T* for long-chain conditions [9], it came to our attention that the Arrhenius expression we used for *fk*_d_ (AIBN) was sub-optimal [41]. This is illustrated in Figure 3, which is an Arrhenius plot showing the *fk*_d_ [8] from Berger [42] that we previously used [9], *k*_d_ for AIBN from an AkzoNobel catalogue [33], and te literature values of *k*_d_ [43,44,45,46,47,48,49,50,51,52] and *fk*_d_ [53] for AIBN in various media. Despite the large number of different solvent media, it is evident from Figure 3 that the AkzoNobel Arrhenius parameters provide a highly accurate description of the literature data for *k*_d_ (AIBN). Because of this, and because one would expect a supplier to have characterized their product meticulously, we have switched to using the AkzoNobel *k*_d_ values in the present work (see Table 2). Furthermore, the data of Figure 3 give confidence that the AkzoNobel fit will hold well in the TFT- and EBz-dominated monomer solutions of our work because it shows at most a minor variation in *k*_d_ amongst solvents of this type.

**Figure 4:**
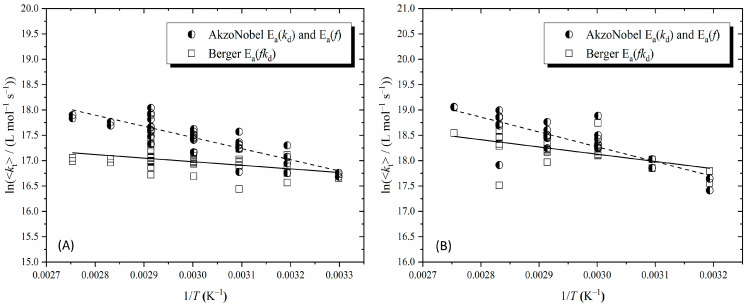
Arrhenius plots for the variation in the overall termination rate coefficient, <*k*_t_>, with temperature, *T*, for AIBN-initiated bulk polymerisations of MMA (**A**, left) and ST (**B**, right). Squares: previously reported values [9] obtained using *fk*_d_ from Berger [42]; half-filed circles: updated values using *fk*_d_ from Table 2; lines: best fits for each data set.

In our prior study [9], we used the Berger results [42] because they account for both AIBN decomposition and efficiency under actual polymerization conditions. These *fk*_d_ values turn out to be in precise agreement with the product of *f* [34] and *k*_d_ [33] from Table 2 at 40 °C, as well as the Fukuda et al. [53] values at the same temperature. It was for this reason that the Berger results—for which *E*_a_(*fk*_d_) = 123.5 kJ mol^−1^—were adopted at all *T* values. However, Table 2 makes clear the problem with this, for it gives *E*_a_(*fk*_d_) = (130.2 + 5.7) = 135.9 kJ mol^−1^. While this change in *E*_a_ may look to be relatively minor (e.g., it can hardly be discerned in Figure 3), it turns out to be highly significant for *E*_a_(<*k*_t_>). This is because Equation (5) shows that *E*_a_(<*k*_t_>) is a balance of different *E*_a_, giving a final result that is much smaller than *E*_a_(*fk*_d_), and on which scale the difference of 12.4 kJ mol^−1^ has a large impact. This is illustrated in Figure 4, which shows our previous <*k*_t_> results as published [9] and as re-analyzed with the *fk*_d_ of the present work, which we believe to more accurate. Because *E*_a_(*fk*_d_) is larger, the obtained *E*_a_(<*k*_t_>) are larger, rising from 6 [9] to 18 kJ mol^−1^ for MMA and from 12 (previously misreported as 14 [9]) to 24 kJ mol^−1^ for ST. These updated values, which we regard as more accurate, will be dissected in the following section. Figure 4 makes clear that the higher *E*_a_(<*k*_t_>) arise because previously we used *fk*_d_ that were too low at high *T*, and hence the obtained <*k*_t_> at these *T* values were too low. The important lesson from this re-analysis is that the accuracy of input data can be of utmost importance in the measurement of trends in <*k*_t_>, such as its *E*_a_.

## 4. Discussion

### 4.1. Theoretical Framework

Although, unfortunately, the following equation [10,54] is not widely appreciated, it is a tremendous tool for understanding RP kinetics [4,55]:

(8) $$\langle k_{t}\rangle =k_{t}^{1,1}{\left[Г\left(\frac{2}{2-e}\right)\right]}^{-2}{\left[\frac{{\left(2R_{i}k_{t}^{1,1}\right)}^{0.5}}{k_{p}c_{M}}\left(\frac{2}{2-e}\right)\right]}^{2e/\left(2-e\right)}$$

In this equation, Γ is the gamma function, *R*_i_ = 2*fk*_d_*c*_I_ is the rate of initiation, and all other parameters have been previously introduced. In particular, the *e* is that of Equation (3), which is the homotermination model used in deriving Equation (8). Also assumed are steady-state, long chains, negligible chain transfer, and the so-called geometric-mean model for *k*_t_*^i,j^* [4]. All these assumptions are necessary; otherwise, a closed expression for <*k*_t_> is not possible. While this list may seem highly restrictive, in fact, steady state and long chains are assumptions that are standardly met, and we have already discussed how our experimental conditions were designed to result in negligible dead-chain formation by transfer. So, attention is speicifically focussed on the simple power-law model for *k*_t_*^i,i^*, Equation (3), and on the geometric-mean model for *k*_t_*^i,j^*. Neither of these are physically realistic [4,5]. However, the latter turns out to be of no consequence, because it has been shown that the trends in Equation (8) are quantitatively accurate regardless of the cross-termination model [56]. With the composite model, in Equation (2), the above result is not strictly valid, which is unfortunate. However, no analytic expression for <*k*_t_> is possible with Equation (2) [10], leaving Equation (8) as the only option for gaining insight. Happily, it was shown that if *DP*_n_ » *i*_c_, then Equation (8) with *e* = *e*_L_ is accurate, and alos analogously, when the average chain size is very short [10], results which make intuitive sense.

In consequence of all this, Equation (8) is a powerful lens for the analysis of RP kinetic data. For example, it has been successfully used to quantitatively explain deviations from the classical rate law for RP that are due to variations of <*k*_t_> with *c*_I_ and *c*_M_ [4,9]. Apropos of which, it is clear from Equation (8) that if one is seeking to understand the variation in <*k*_t_> with *T*, then a sensible experimental design involves keeping both *c*_I_ and *c*_M_ constant, because this eliminates two of the many factors that cause <*k*_t_> to vary. This is why we kept these concentrations constant across all temperatures in our experiments (see Table 1), and the fact that workers have not historically paid attention to this is one of several reasons for the notorious scatter in Arrhenius plots of <*k*_t_> found in the literature [6,9], making it almost impossible to identify trends in *E*_a_(<*k*_t_>).

Given constant *c*_I_ and *c*_M_, one has from Equation (8) that

(9) $$E_{a}\left(\langle k_{t}\rangle \right)=\left(1+a\right)E_{a}\left(k_{t}^{1,1}\right)+aE_{a}\left(f\right)+aE_{a}\left(k_{d}\right)-2aE_{a}\left(k_{p}\right),where\;a=\frac{e}{2-e}$$

This equation is a paradigm-shifting result, since it indicates that *E*_a_(<*k*_t_>) is not just a function of the activation energy of a termination process, viz. *E*_a_(*k*_t_^1,1^), but, due to CLDT, it is also greatly affected by how the rates of initiation and propagation vary with temperature because these processes change the RCLD, which then changes <*k*_t_>, as earlier explained using Equation (1). Thus, one sees the power of Equation (8); it furnishes a relatively simple quantitative means for analyzing *E*_a_(<*k*_t_>) values, viz. Equation (9). This is as opposed to large-scale kinetic simulations, the complexity of which tends to obscure any simple patterns of behaviour in the output. For these reasons, we will use Equation (9) to interrogate our kinetic data.

**Figure 5:**
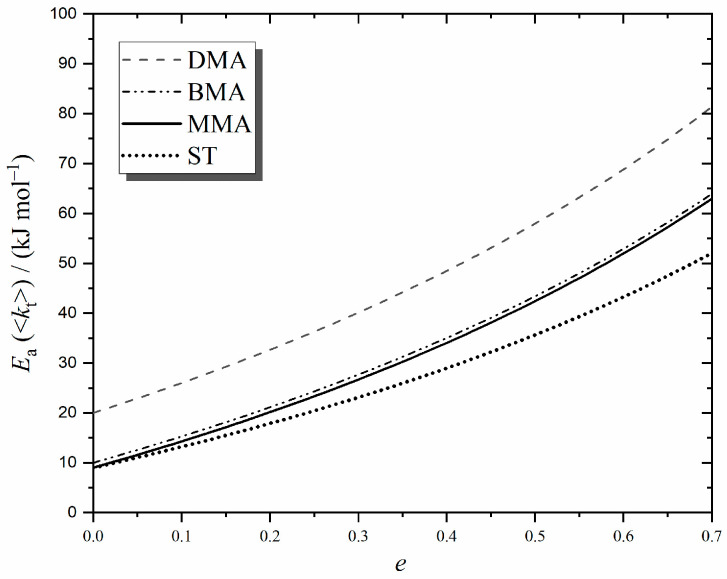
Predicted activation energy of the overall termination rate coefficient, *E*_a_(<*k*_t_>), for steady-state MMA, BMA, DMA, and ST polymerization as a function of *e*, the strength of the chain-length dependence of termination (under conditions of constant *c*_M_, constant *c*_AIBN_ and negligible transfer).

We start with Figure 5, which presents evaluations of Equation (9) for the four monomers of this work using the parameter values of Table 2, all of which are based on the most accurate experimental data available. As shown, *E*_a_(<*k*_t_>) = *E*_a_(*k*_t_^1,1^) for chain-length-independent termination, *e* = 0, as expected. However, the interesting thing is that *E*_a_(<*k*_t_>) ≠ *E*_a_(*k*_t_^1,1^) when there is any CLDT (*e* > 0). Specifically, *E*_a_(<*k*_t_>) increases as the chain-length dependence of termination increases. Furthermore, the variation with *e* depends on the monomer type, e.g., compare ST with the methacrylates in Figure 5. From Equation (9), it is clear that this is due to *E*_a_(*k*_p_) being different for ST and the methacrylate family (see Table 2), which evidently means that ST’s RCLD changes differently as *T* is varied. These findings immediately explain the variability and apparent lack of pattern in the literature values of *E*_a_(<*k*_t_>) and, in particular, their common inequality with *E*_a_ of diffusion processes. Even the methacrylates, which often show family-type commonality in *E*_a_, can be seen from Figure 5 to have different *E*_a_(<*k*_t_>) for a particular *e*, this stemming from *E*_a_(*k*_t_^1,1^) variation, e.g., this is why the results for DMA stand apart—due to its large size and high viscosity, this monomer has a significantly higher *E*_a_(*k*_t_^1,1^) than MMA and BMA (see Table 2).

### 4.2. Long Chains

In our previous work [9], we carried out bulk polymerisations of ST and MMA, as opposed to the relatively dilute-solution (*c*_M_ = 0.67 mol L^−1^) polymerisations of the present work. Furthermore, we used *c*_AIBN_ = 0.0005, 0.005, and 0.05 mol L^−1^, as opposed to only the highest of these values here. For these reasons, our *DP*_n_ values were much larger in our previous work, as per Equation (7) with higher *c*_M_ and lower *c*_I_. Specifically, we had *DP*_n_ » *i*_c_. This means that one would expect <*k*_t_> behaviour to be dictated by Equation (2b), i.e., the long-chain portion of the composite model. For *E*_a_(<*k*_t_>), one may use Equation (9) to see whether this is the case. It rearranges to

(10) $$e=\frac{2a}{1+a},\;where\;a=\frac{E_{a}\left(\langle k_{t}\rangle \right)-E_{a}\left(k_{t}^{1,1}\right)}{{E_{a}\left(k_{t}^{1,1}\right)+E}_{a}\left(f\right)+E_{a}\left(k_{d}\right)-2E_{a}\left(k_{p}\right)}$$

This equation provides a novel and relatively easily executed procedure for obtaining the chain-length dependence of termination from steady-state measurements of *E*_a_(<*k*_t_>), where we stress that such measurements must be carried out with constant *c*_M_, constant *c*_I_, and the same initiator.

Using Equation (10) with the values of Table 2, our long-chain MMA value of *E*_a_(<*k*_t_>) = 18 kJ mol^−1^ from Figure 4A gives *e* = 0.17, which is in remarkably precise agreement with experimentally measured [4,24] values and with the theoretically predicted [4,57] value of *e*_L_ = 0.16. For ST, the situation is not quite so remarkable, with the long-chain value of *E*_a_(<*k*_t_>) = 24 kJ mol^−1^ from Figure 4B giving *e* = 0.31, which is above the theoretical prediction for *e*_L_ (also 0.16), as well as other experimental measurements (*e*_L_ ≈ 0.2 [4]). This may be due to the low *k*_p_ and high <*k*_t_> of ST systems, both of which result in smaller *DP*_n_ values (see Equation (7)) that are closer to the crossover chain length *i*_c_. Hence, there may be some influence from smaller chains, for which *e* = *e*_S_ is higher than *e*_L_ (see Section 1), thus making *E*_a_(<*k*_t_>) higher (see Figure 5). Another thing to note is the influence of *E*_a_(*k*_t_^1,1^). For example, if, instead of 9 kJ mol^−1^ (Table 2), one uses the value 11 kJ mol^−1^ in Equation (10), as measured for toluene diffusion coefficients [58] and for styrene fluidity [26], one obtains *e* = 0.27 for ST, which is closer to the known *e*_L_. If nothing else this illustrates just how sensitive the value of *E*_a_(<*k*_t_>) is to many underlying factors.

The higher than expected value of *E*_a_(<*k*_t_>) for ST should not obscure two really important and novel accomplishments from the preceding paragraph of work: (1) the measurement of *E*_a_(<*k*_t_>) > *E*_a_(*k*_t_^1,1^) for steady-state polymerization has been fully explained in terms of CLDT; and (2) experimental values of low-conversion *E*_a_(<*k*_t_>) have been shown to be in broad quantitative agreement with what CLDT predicts for long chains (see Figure 5 with *e* ≈ 0.2).

It is worth mentioning that our long-chain *E*_a_(<*k*_t_>) for MMA and ST are actually very close to the values obtained from considering the large number of <*k*_t_> values from the literature that are presented in our previous work [9]: Figure S1 yields *E*_a_(<*k*_t_>) = 14 kJ mol^−1^ for literature MMA, as compared with 18 kJ mol^−1^ here, while for ST (Figure S2), these values are 21 and 24 kJ mol^−1^, respectively. Of course, the literature <*k*_t_> values are highly scattered, and the underlying experiments involved a wide variety of initiators, *c*_I_ and *c*_M_, as opposed to the deliberate design of our experiments recognizing the impacts of CLDT (see above). However, one can expect effects from such variability to cancel out when <*k*_t_> values from multiple and diverse data sets are combined and fitted, which evidently is the case. This provides enhanced confidence in our experimental procedures, and thus the interpretations attributed to our results.

Lastly, there is something important about the results of this section that may easily go unappreciated. It is that the theory behind Equation (8) and the value *e*_L_ ≈ 0.2 are established beyond doubt [4], and hence, there is no conjecture about the finding *E*_a_(<*k*_t_>) ≈ 20 kJ mol^−1^ for the chemically initiated, long-chain polymerization of MMA, ST, and other similar monomers (see Figure 5). Notably, this value is approximately double that expected from *E*_a_(*k*_t_^1,1^), i.e., by viewing *E*_a_(<*k*_t_>) as arising purely from diffusion. We contend that this is an important paradigm shift in understanding and experimentally addressing RP kinetics.

### 4.3. Short Chains

Figure 5 shows that *E*_a_(<*k*_t_>) is expected to increase as CLDT becomes stronger. How can this be tested? One cannot just dial up the *e* of a system in the way that one can choose a value of *c*_M_ or *c*_I_, for example. However, if one shifts the RCLD to short enough chain lengths, then Equation (2a) should start to play a more prominent role in determining <*k*_t_> behaviour, and because *e*_S_ > *e*_L_, one should therefore observe higher *E*_a_(<*k*_t_>). That is why we designed our present experiments to have low *c*_M_ and relatively high *c*_I_, thus producing chains with low *DP*_n_, in many cases, far less than *i*_c_ (see Table 1). As far as we are aware, ours is the first deliberate determination of *E*_a_(<*k*_t_>) for thermally induced polymerization in the short chain-length regime, *DP*_n_ ≤ *i*_c_. That this was achieved by using a high level of solvent is not of mechanistic consequence for termination, because bulk polymerizations at very low conversion—as considered in the preceding section—also have dilute-solution conditions, where the solvent is the monomer. Thus, termination in each case is the same mechanistically, meaning that *e*_S_ and *e*_L_ are much the same.

**Figure 6:**
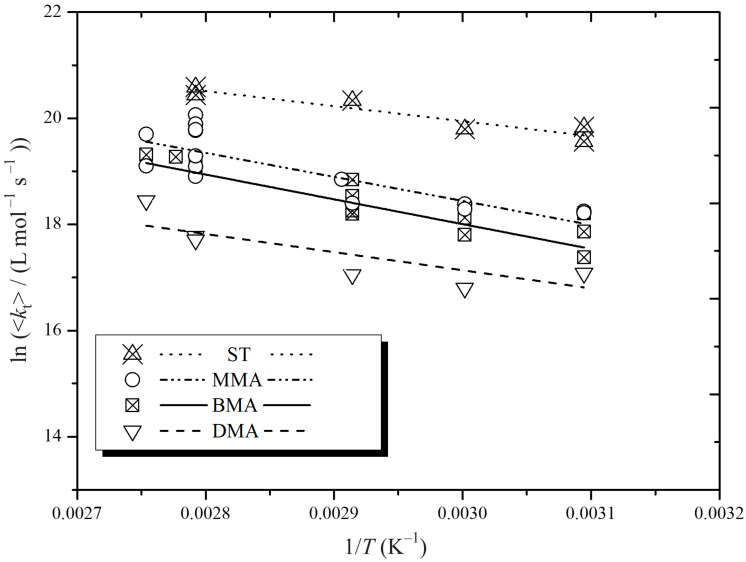
Arrhenius plot for the variation in the overall termination rate coefficient, <*k*_t_>, with temperature, *T*, for the AIBN-initiated, dilute-solution polymerization of ST, MMA, BMA, and DMA, as indicated. Points: experimental values from Table 1; lines: best fits for each data set.

Our <*k*_t_> values for AIBN-initiated experiments are presented in Figure 6. We do not include the relatively small number of BTMHP data (see Table 1 and Figure 2) because we are investigating *E*_a_(<*k*_t_>), which Equation (9) reveals as depending on the initiator. So, by restricting our analysis to AIBN results, we are controlling the number of variables as much as possible, just as with our use of constant *c*_I_ and *c*_M_ across all *T*.

There are several ways in which the results of Figure 6 are qualitatively pleasing: (1) Our MMA and ST results of Figure 6 are clearly higher in value than those of Figure 4 at the same *T*. This is plainly a manifestation of CLDT: smaller *DP*_n_ results in higher <*k*_t_>. (2) The stark trend <*k*_t_>(ST) > <*k*_t_>(MMA) is also most plausibly explained as originating in CLDT [8,9]: the diffusion coefficients of MMA and ST are very similar, which means their *k*_t_^1,1^ values will also be very similar, and hence, their difference in <*k*_t_> likely stems from ST’s slower propagation, which results in its RCLD being more weighted towards small chains and therefore <*k*_t_> being higher, as captured in Equation (8). (3) This reasoning also helps to explain the observation, as also made for longer chains [59], that <*k*_t_>(MMA) > <*k*_t_>(BMA) > <*k*_t_>(DMA), for *k*_p_ increases in this order for these monomers (see Table 2). However, there is also a reinforcing effect here from *k*_t_^1,1^ because it becomes smaller as the methacrylate size becomes bigger. (4) The *E*_a_(<*k*_t_>) values derived from Figure 6, which are presented in Table 3, are indeed higher than those observed for long chains, as discussed in the previous section. This is consistent with the expectation from the composite model.

Before proceeding, it is important to stress that none of the trends identified in the preceding paragraph can be an artefact of *f* and *k*_d_ values because all data analysis has been with identical *f*(*T*) and *k*_d_(*T*): were these values changed, then all <*k*_t_> values would be identically changed, and thus all the noted trends would still be obtained. Furthermore, we do not believe that any of these trends are artefacts of noise in our data because it seems clear from Figure 4 and Figure 6 that the quantitative differences underlying these trends are larger than the experimental error.

Turning now to quantitative analysis, we have applied Equation (10) to our *E*_a_(<*k*_t_>) values and listed the resulting values of *e* in Table 3. Importantly, all are between *e*_S_ and *e*_L_ in value, as one would expect. The *e* obtained for MMA and BMA are rather amazing in that they are only marginally below the range *e*_S_ = 0.5–0.65, which is what has been obtained in experimental measurements of *k*_t_*^i,i^* for methacrylates [4,36], with such values being held to arise from centre-of-mass diffusion being the rate-determining step for termination [4,10]. Of course, one would not expect to obtain *e* values quite this large in the present experiments because *DP*_n_ are not so small that the entire RCLD has *i* < *i*_c_, and thus there will still be some long-chain influence on the obtained value of *e*. Hence, one can take our MMA and BMA results as being fully consistent with composite-model parameter values, and so the perhaps surprisingly high *E*_a_(<*k*_t_>) values are resoundingly explained.

While the DMA and ST results of Table 3 are at least qualitatively in line with the notions of the previous paragraph (higher *e* and elevated *E*_a_(<*k*_t_>)), it must equally be admitted that the quantitative boosts are not as large as anticipated. Plausible explanations for this may be offered. In the case of DMA, *i*_c_ is in the range 50–70, and so it is evident from Table 1 that the accessed *DP*_n_ values simply do not meet the criterion of being far less than *i*_c_ in value. This is simply because the high *k*_p_ and low <*k*_t_> of DMA mean that it is not possible to achieve *DP*_n_ < *i*_c_ across the temperature range of this work with the same *c*_M_ and *c*_I_ as in other systems. Consequently, one would expect the behaviour of DMA in our present experiments to be far more dictated by the long-chain value *e* ≈ 0.16 (see above). Furthermore, the value of *e* obtained from *E*_a_(<*k*_t_>) is dependent on the value of *E*_a_(*k*_t_^1,1^), for which the one available measurement of 20 kJ mol^−1^ is rather high compared with other methacrylate values (see Table 2). If, for example, one changes this to 15 kJ mol^−1^, then *e* = 0.27 is obtained with Equation (10).

With regard to ST, the first thing to say is that our observation of *E*_a_(<*k*_t_>,MMA) > *E*_a_(*k*_t_,ST) is at least consistent with Figure 5, where it stems from *E*_a_(*k*_p_) being higher for ST. However, it is equally clear from Figure 5 that one does not expect the difference to be as large as 38 vs. 25 kJ mol^−1^, respectively. One explanation for this could be a contribution in the case of ST from the transfer to EBz. This solvent was employed because it is the saturated analogue of ST, and thus it furnishes a solution environment that is nearly physically identical to bulk ST. However, EBz has C–H bonds, which are labile in terms of chain transfer. On the other hand, the solvent used for methacrylate work, TFT, does not. Indeed, in previous work, we have demonstrated via ESI-MS that transfer to methyl isobutyrate definitely occurs when it is used as a solvent in MMA polymerization, whereas this reaction is eliminated when TFT is used instead [41]. If any transfer to EBz did occur in our ST experiments, it would have elevated the value of <*k*_t_> by generating more short radicals. This effect would be more pronounced at lower temperatures due to the much stronger decline in *R*_i_ with temperature, meaning that the ‘competition’ between transfer and initiation in dead-chain formation somewhat evens out [7]. This would explain that our measured *E*_a_(<*k*_t_>) is lower than expected on the basis of dead-chain formation by termination alone.

Another observation about our ST experiments is that the ESI-MS analysis of the generated polymer indicated the presence of O_2_ in some chains—see Figure S3. This was not found with methacrylate systems [41], and even with ST, it was a surprise because there was no evidence of inadequate deoxygenation in our *x*(*t*) measurements in the form of inhibition (delayed polymerization start) or retardation (rate acceleration as residual O_2_ is consumed). It is also not clear why a deoxygenation procedure that worked perfectly adequately for the three methacrylates should not have been so effective for ST, unless there were high levels of O_2_ in the EBz. Furthermore, it is clear from Figure S3 that some O_2_ was present in our ST experiments, and this must have given rise to an elevation of <*k*_t_>. Since O_2_ solubility increases as *T* decreases, this retardation effect must have been greater at lower *T* values, which also plausibly explains *E*_a_(<*k*_t_>) being slightly lower than expected.

Although these potential issues with our ST systems are not ideal, it seems helpful for other workers that we draw attention to them.

### 4.4. Investigations Involving Degree of Polymerization

A CLDT relationship that is simpler and therefore better known than Equation (8) is

<*k*_t_> = *G k*_t_^1,1^ *DP*_n_^−*e*^ (11)

Although this equation is challenging to derive [10,54], it is intuitive in that it says that if *i* = *DP*_n_ is used in Equation (3), then the *k*_t_*^i,i^* obtained is essentially equal to <*k*_t_>. This is because the value of *G*, which depends only on *e* and *λ*, is close to 1 [10,54]. The assumptions behind Equation (11) are the same as those for Equation (8), except for one important difference: Equation (11) holds also when there is dead-chain formation by transfer [7,56]. This confers on it the additional advantage of being more widely applicable. A further advantage in this regard is that variables such as *c*_M_ and *c*_I_ are not explicitly present in Equation (11) because they affect both <*k*_t_> and *DP*_n_ in ways that are implicitly captured by the relationship. This means that one does not need to be careful about controlling these variables in a set of experiments.

Due to these advantages, Equation (11) has been the most commonly used vehicle for probing CLDT [4]. Mostly, this has been carried out via the log–log plotting of simultaneously determined <*k*_t_> and *DP*_n_ data, with the slope of the plot yielding *e* and the intercept *k*_t_^1,1^. Such experiments have always been carried out at a constant temperature. But, because *T* is the variable of interest in the present work and because Equation (11) must also hold as *T* is varied, a new way of looking at it is prompted, for in terms of *E*_a_, it yields

*E*_a_(<*k*_t_>) = *E*_a_(*k*_t_^1,1^) − *e* × *E*_a_(*DP*_n_) (12)

This result assumes negligible variation in G and *e* with temperature. The former is reasonable because, as mentioned, *G* is always close to 1 in value. The latter finds experimental support from the many SP-PLP-EPR investigations that have been carried out, for they always find negligible variation in *e*_S_ and *e*_L_ with *T* [14,24,26,36].

It is well known that *DP*_n_ decreases as *T* increases in chemically initiated RP, meaning *E*_a_(*DP*_n_) < 0. For *e* = 0 (chain-length-independent termination), Equation (12) shows that this has no effect on *E*_a_(<*k*_t_>), which is simply equal to *E*_a_(*k*_t_^1,1^), as it must be. However, for the reality of CLDT (*e* > 0), *E*_a_(*DP*_n_), < 0 means *E*_a_(<*k*_t_>) > *E*_a_(*k*_t_^1,1^), which is fully consistent with Figure 5, our experimental results, and the new understanding of *E*_a_(<*k*_t_>) that we have herein developed. It seems worthwhile to push the envelope and see if Equation (12) is quantitatively successful. To do this, we must introduce our measurements of molar mass, *M*.

**Figure 7:**
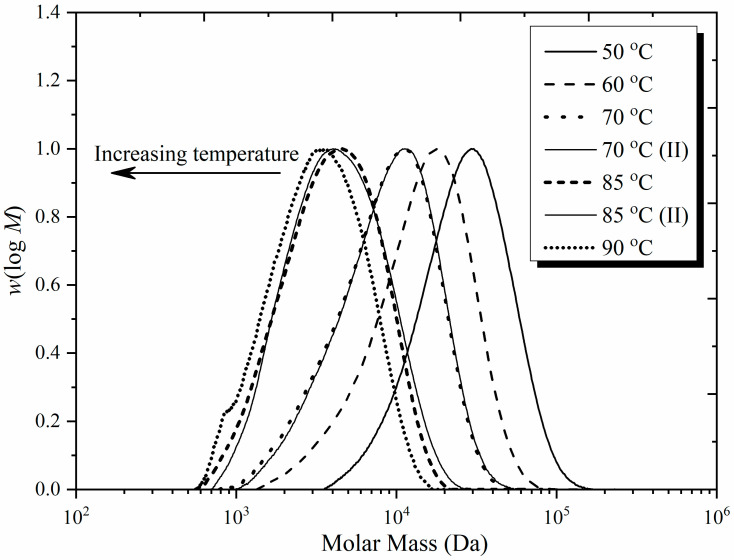
SEC distributions of polymer from MMA polymerizations at various temperatures, as indicated, with *c*_M_ = 0.67 mol L^−1^, TFT as solvent, and *c*_AIBN_ = 0.05 mol L^−1^. Distributions are presented as *w*(log*M*), where *w* is weight fraction and *M* is molar mass. For ease of comparison, all peak heights have been set equal to 1.

Figure 7 presents SEC results for our MMA experiments. It is evident that reproducibility is good and that *M* decreases with increasing *T*, as expected. The corresponding values of *DP* are in line with expectations (see Table 1) when it is remembered that (1) the SEC peak is close in value to *DP*_w_, the weight-average *DP*, which will be about double the value of *DP*_n_ [60], and (2) the effect of chain-length-dependent propagation, which has been ignored in using Equation (7), will be to add about 3–5 to the value of *DP*_n_ [61,62] because of the initial rapid growth of chains. Thus, for example, the 90 °C MMA value of *DP*_n_ ≈ 10 in Table 1 is consistent with peak *DP* ≈ 25 for this system in Figure 7. These are only back-of-the-envelope calculations and should not be taken literally—they are merely to establish that there is decent agreement between our SEC results and what we expected for *DP*_n_.

**Figure 8:**
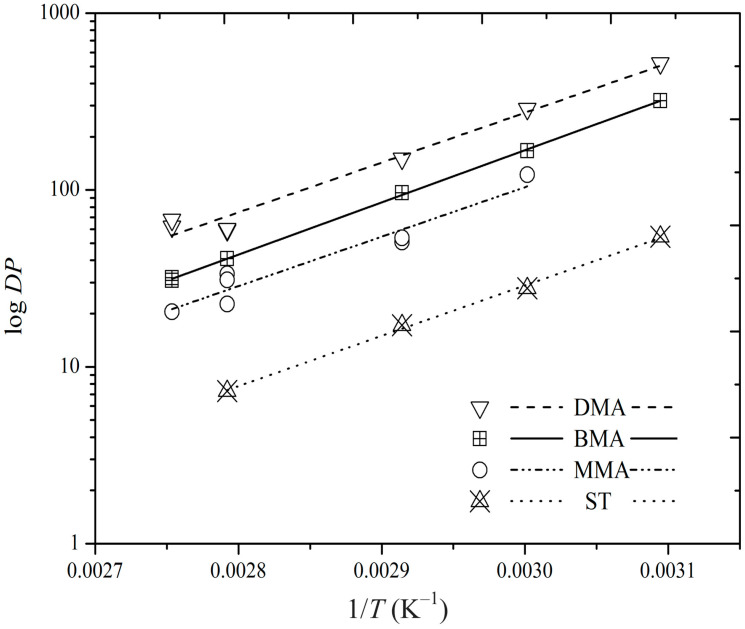
Arrhenius plot for variation in the peak degree of polymerization, *DP*, with temperature, *T*, for AIBN-initiated, dilute-solution polymerizations of ST, MMA, BMA, and DMA, as indicated. Points: experimental values; lines: best fits for each data set.

Values of *DP*_p_, the peak *DP*, from all our SEC measurements are presented in Figure 8. We opted to use *DP*_p_ rather than *DP*_n_ for quantitative analysis because the latter is prone to significantly higher error than the former due to the difficulty of converting *w*(log*M*) into accurate number-molar mass distribution, *n*(*M*), at very low *M*, where *w*(log*M*) is small in value but *n*(*M*) is large. Of course, Equation (11) calls for *DP*_n_, but recall that our interest is in *E*_a_(*DP*_n_), which is obtained from the variation in log(*DP*_n_) with *T*^−1^. This variation will be the same as that for log(*DP*_p_), providing that *DP*_p_ ~ *DP*_n_, which should be the case to good approximation. We note that in previous work investigating Equation (7), it was shown that the approach of using *DP*_p_ is indeed more reliable [60].

All systems in Figure 8 show decreasing *DP*_p_ with increasing *T*, as expected (see above). This gives rise to negative values of *E*_a_(*DP*_p_), as tabulated in Table 4. It is evident that values for all four monomers are comparable, as is also visually apparent from Figure 8. Note, however, that the absolute values of *DP*_p_ show the expected variation from monomer to monomer, with DMA > BMA > MMA > ST, due to *k*_p_ decreasing in the same order (see Table 2) and <*k*_t_> increasing in this order (see Figure 6).

How can the *E*_a_(*DP*_p_) values of Table 4 be put to use? If *E*_a_(<*k*_t_>) is not known, then it could be estimated using Equation (12) with *E*_a_(*DP*_p_) ≈ *E*_a_(*DP*_n_) combined with known values of *e* and *E*_a_(*k*_t_^1,1^), an approach with the advantage of requiring less input data than that of Equation (9). For example, using *E*_a_(*k*_t_^1,1^) from Table 2 together with *e* ≈ 0.6 for short-chain MMA [24], one obtains *E*_a_(<*k*_t_>) = 40 kJ mol^−1^, which is very close to the value measured here for MMA, viz. 38 kJ mol^−1^ (see Table 3). Equally, from measured *E*_a_(<*k*_t_>), one could estimate *E*_a_(*DP*_n_). Alternatively, where both *E*_a_(<*k*_t_>) and *E*_a_(*DP*_p_) ≈ *E*_a_(*DP*_n_) have been measured from the same experiments, one can obtain an estimate of *e* for the prevailing conditions using the following rearranged from of Equation (12):

(13) $$e=\frac{E_{a}\left(\langle k_{t}\rangle \right)-E_{a}\left(k_{t}^{1,1}\right)}{-E_{a}\left({DP}_{n}\right)}$$

This approach is the varying-*T* analogue of the aforementioned approach of plotting log(<*k*_t_>) versus log(*DP*_n_), noting that the latter approach assumes constant *k*_t_^1,1^ and thus can only be used for constant-*T* data. Equation (13) is a curious result in that it sets no constraint on the value of *E*_a_(*DP*_n_) when termination is chain-length-independent (*e* = 0, meaning *E*_a_(<*k*_t_>) = *E*_a_(*k*_t_^1,1^)), but when *e* ≠ 0, this equation rigidly stipulates the relationship between these three activation energies, even though *E*_a_(*DP*_n_) must also be consistent with the Mayo equation (Equation (7)).

Table 4 also presents values of *e* obtained using Equation (13) with *E*_a_(*k*_t_^1,1^) from Table 2, *E*_a_(<*k*_t_>) from Table 3, and *E*_a_(*DP*_n_) = *E*_a_(*DP*_p_) from Table 4. Not surprisingly the results are a mixed bag, exactly along the lines of the earlier *E*_a_(<*k*_t_>) results, and for the same reasons. Namely, for MMA and BMA, the obtained *e* values are in very good agreement with expectation for *e*_S_, while for DMA and ST, the obtained values are somewhat lower. For DMA, this is plausibly due to the chains being more in the long-chain regime, consistent with Figure 8. Indeed, it would be interesting to investigate *E*_a_(*DP*_n_) for long chains, as discussed further in the following section. The purpose of the present section has primarily been to draw attention to the novel result that is Equation (12), discussing what it means and illustrating how it can be used with experimental data.

### 4.5. Further Consequences and Future Avenues

The results of this paper make many predictions worthy of further experimental investigation. Some examples that occur are as follows:

(i) ***E*_a_(<*k*_t_>) for long-chain methacrylates**. The predictions of Figure 5 for long-chain (*e* ≈ 0.2) systems have been probed only for ST and MMA in this work. It obviously would be of interest to extend this to other methacrylates, e.g., BMA and DMA. This requires experimental design as in the present work, viz. constant *c*_M_, *c*_I_, and initiator. Of particular interest is whether *E*_a_(<*k*_t_>) ≈ 32 kJ mol^−1^ for long-chain DMA (see Figure 5) on account of *E*_a_(*k*_t_^1,1^) ≈ 20 kJ mol^−1^. Indeed, we note that if the value of *e* is confidently known, then a measurement of *E*_a_(<*k*_t_>) can be used to estimate the value of *E*_a_(*k*_t_^1,1^).

(ii) ***E*_a_(*DP*_n_) for long chains**. Equation (12) rearranges to

*E*_a_(*DP*_n_) = [*E*_a_(*k*_t_^1,1^) − *E*_a_(<*k*_t_>)]/*e* (14)

**Figure 9:**
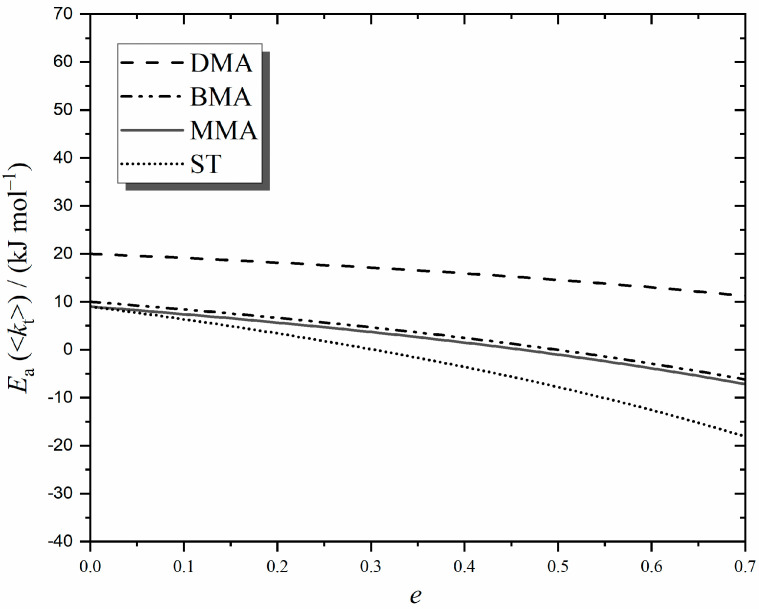
As for Figure 5, except that *E*_a_(*k*_d_) = 0 in order to mimic photoinitiation.

It has been seen in this work that *E*_a_(*k*_t_^1,1^) ≈ 10 kJ mol^−1^, *E*_a_(<*k*_t_>) ≈ 20 kJ mol^−1^, and *e* ≈ 0.2 for long-chain MMA. Using these in Equation (14) predicts *E*_a_(*DP*_n_) ≈ −50 kJ mol^−1^ for chemically initiated, long-chain MMA. Is this the case? Such predictions could be investigated, whether approximately (as in this example) or precisely, noting that <*k*_t_> and *DP*_n_ measurements from the same experiments are required, as opposed to picking values from separate experiments out of the literature.

(iii) **Polymerization with continuous photoinitiation**. Chemical initiation relies on thermal energy to bring about the homolysis of the initiator, and thus the process has a high *E*_a_. With photoinitiation, the homolysis is induced by incident radiation, and hence the rate of primary radical generation will be independent of temperature, to good approximation at least. Thus, with (continuous) photoinitiation, one should switch to using *E*_a_(*k*_d_) = 0 in Equation (9). For this reason, we have carried out evaluations of this equation in which all else is the same as before (i.e., the values of Table 2 are used) aside from *E*_a_(*k*_d_). The results are presented in Figure 9. Of course, the value of *E*_a_(*f*) for a photoinitiator will not be the same as for AIBN; however, there will still be initiator inefficiency that can be expected to vary with *T* in the same qualitative manner as for AIBN. Furthermore, this value has only a very minor effect on results. For these reasons, it seems appropriate to stick with the Table 2 value of *E*_a_(*f*) for the present illustrative purposes.

The remarkable effect seen in Figure 9 is that *E*_a_(<*k*_t_>) with photoinitiation is predicted to decrease with increasing *e*, opposite to what is observed in Figure 5 for chemical initiation. This is because initiation no longer has a strongly temperature-dependent effect on the RCLD, and thus other factors play a more noticeable role. Specifically, increasing *k*_p_ weights the RCLD towards longer chains, an effect which counterbalances that of increasing *k*_t_*^i,j^*. Indeed, Figure 9 shows that at high enough *e*, this effect of *k*_p_ becomes so strong that *E*_a_(<*k*_t_>) will become negative in value for monomers like ST and MMA, as first pointed out long ago [8].

Obviously, it would be of interest to investigate the predictions of Figure 9 by measuring *E*_a_(<*k*_t_>) for polymerizations with continuous photoinitiation. Encouragement can be found in the literature in the form of *E*_a_(<*k*_t_>) = 5.6 kJ mol^−1^, having been obtained for MMA in SP-PLP experiments [63]. Remarkably, 5.6 kJ mol^−1^ is exactly what Figure 9 predicts for long-chain (*e* ≈ 0.2) MMA! However, this result should be taken as being indicative only because the photoinitiation in SP-PLP is not continuous. That said, the Olaj group conducted a lot of theoretical work showing that trends in <*k*_t_> measured by PLP are the same as those with continuous initiation [64], so there is good reason to think that Buback and Kowollik’s SP-PLP result should reflect what would be found with continuous photoinitiation.

**Figure 10:**
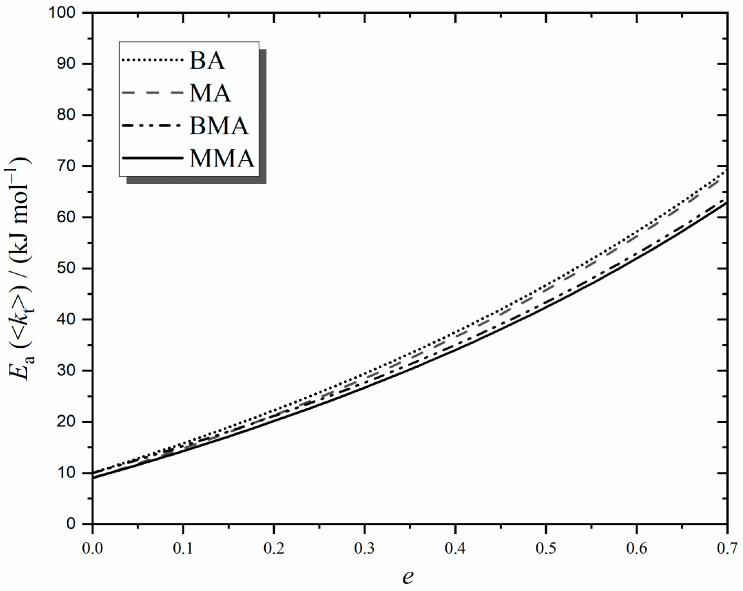
As for Figure 5, except that *E*_a_(*k*_p_) = 17.3 kJ mol^−1^ [65] was used to obtain methyl acrylate (MA) estimates, and *E*_a_(*k*_p_) = 17.9 kJ mol^−1^ [66] for butyl acrylate (BA).

An important take-home message here is that *E*_a_(<*k*_t_>) measured with laser-based techniques will be significantly lower than when applied for chemical initiation. Specifically, photoinitiation will result in *E*_a_(<*k*_t_>) < *E*_a_(*k*_t_^1,1^) (see Figure 9), while for chemical initiation, one has *E*_a_(<*k*_t_>) > *E*_a_(*k*_t_^1,1^) (Figure 5).

(iv) **Acrylate polymerization**. In terms of termination kinetics, acrylates differ from methacrylates in two major ways: they have lower *E*_a_(*k*_p_) and higher *e*_S_. Specifically, *E*_a_(*k*_p_) = 17.3 and 17.9 for chain-end methyl acrylate (MA) [65] and butyl acrylate (BA) [66], respectively, while *e*_S_ ≈ 0.8 for these monomers [67]. Both these effects serve to elevate *E*_a_(<*k*_t_>) in chemically initiated polymerization, as can be seen in Figure 10. This shows evaluations of Equation (9) for MA and BA with the just-given *E*_a_(*k*_p_) but with all else, as in Table 2 for MMA and BMA, respectively. Of course, *E*_a_(*k*_t_^1,1^) for an acrylate will be different to that for the corresponding methacrylate, but measurements [67] indicate that these differences are very small (of order 1–2 kJ mol^−1^ at most), which is as one would expect on the basis of small-molecule diffusion, e.g., an MA monomer is physically very similar to MMA. So, for illustrative purposes, it was decided to make *E*_a_(*k*_p_) the only altered value in performing acrylate calculations. For comparison, the MMA and BMA results of Figure 5 are also presented in Figure 10.

Figure 10 makes the prediction that *E*_a_(<*k*_t_>) will be slightly higher for long-chain (*e* = *e*_L_ ≈ 0.2 [67]) acrylates than methacrylates. It additionally predicts that this difference will become quite appreciable if very short chain lengths are accessed because of the higher value of acrylate *e*_S_ coming into play. Of course, these findings apply only for very low temperatures, at which a negligible fraction of mid-chain radicals can be expected. Once backbiting becomes significant, the kinetics of acrylate polymerization are greatly complicated [68], and it is not clear what should be expected of *E*_a_(<*k*_t_>) in chemically initiated systems that constantly have both mid-chain and end-chain radical populations present.

(v) **Dominant transfer**. Up until now, this work has assumed dead-chain formation predominantly by termination. The opposite limit to this is dominant transfer [7]. For this situation, the analogue of Equation (9) is [7]

(15) $$E_{a}\left(\langle k_{t}\rangle \right)=E_{a}\left(k_{t}^{1,1}\right)+e{\times E}_{a}\left(C_{trM}+C_{trS}\frac{c_{S}}{c_{M}}\right)$$

Here, the notation of Equation (7) has been used for transfer terms. Note that, once again, there is the phenomenon that *E*_a_(<*k*_t_>) > *E*_a_(*k*_t_^1,1^) due to the RCLD favouring shorter chains—which have higher *k*_t_*^i,j^*—as the temperature increases due to the *C*_tr_ ratios having positive *E*_a_.

It would be of interest to test the predictions of Equation (7). Experiments with a constant ratio of transfer agent to monomer concentration across different *T* values are recommended. If this is the case and there is a dominant transfer with *E*_a_(*C*_tr_) = 20 kJ mol^−1^ (a typical value for transfer to labile solvent [69]), then *E*_a_(*k*_t_^1,1^) = 10 kJ mol^−1^ and *e* = 0.2 (long chains) results in a prediction of *E*_a_(<*k*_t_>) = 14 kJ mol^−1^, while *e* = 0.5 (short chains) results in *E*_a_(<*k*_t_>) = 20 kJ mol^−1^. Note that these values are independent of the rate of initiation, so another interesting prediction is that photoinitiation and chemical initiation should give the same *E*_a_(<*k*_t_>), which is opposite to the situation with the termination control of dead-chain formation (see above). That said, with chemical initiation, it is hard to remain in the limit of dominant transfer as *T* is raised because of *E*_a_(*k*_d_) being so large.

Where there is significant dead-chain formation by both transfer and termination, no analytic expressions exist for <*k*_t_> in the event of CLDT, so all one can say is that *E*_a_(<*k*_t_>) will be in between the two limits of Equation (9) and Equation (15). For this reason, it is advisable to carry out experimental investigations of termination in one of these limits. The parameter values obtained can then be used in computer-based predictions for the situation of mixed termination and transfer. If one tries to investigate termination in this mixed-mode situation, then the only option is computer-based modelling with a plethora of adjustable parameters, a practice which is not ideal if one’s aim is to obtain mechanistic insight.

## 5. Conclusions

Understanding how <*k*_t_> varies with *T* is of utmost importance for predicting how the rate of polymerization and *DP*_n_ vary with *T* in RP. Where a reaction has a complex mechanism, it is well known in physical chemistry that its *E*_a_ may be complicated in that it can be a function of several individual *E*_a_ for elementary steps in the mechanism. However, there is no such expectation for termination in RP because it is a facile, single-step reaction. Prima facie, it is therefore a surprise that no clear understanding of *E*_a_(<*k*_t_>) has emerged over many decades of RP study. In this work, we have addressed this uncomfortable situation, finding that it is explained by the chain-length-dependent nature of termination, which in fact bestows complicated and indeed fascinating behaviour on *E*_a_(<*k*_t_>), as encapsulated in Equation (9). While this result may strike many workers as being overly elaborate and unnecessarily complicated, in fact, it contains only the bare minimum detail, for it ignores chain transfer, chain-length-dependent propagation, and composite-model termination, all of which can exert further influences.

Another objection to Equation (9) is that it applies for the broad RCLDs from continuous initiation, and termination is better studied in designer experiments with narrow RCLD, most notably time-resolved SP-PLP [5]. While this is indeed beyond dispute for obtaining values of *k*_t_*^i,i^*, it does not change that the overwhelming majority of commercial RP is via chemically initiated systems without a reversible-deactivation RP agent. What are *E*_a_(<*k*_t_>) for such systems? They are necessary to know, and a major point of this work has been to make clear that they do not follow in a simple way from *E*_a_(*k*_t_*^i,i^*) measurements. Rather, we have argued that Equation (9) is an invaluable lens for understanding chemically initiated *E*_a_(<*k*_t_>) in all its complexity. We have conducted this by demonstrating that measurements of chemically initiated *E*_a_(<*k*_t_>) using a simple technique are consistent with the predictions of Equation (9) for different monomers and different *DP*_n_. CLDT parameters from SP-PLP-EPR experiments are indispensable inputs in this process, which shows that both types of investigation are essential for establishing a total picture of termination kinetics in RP. We have also pointed out interesting predictions from Equation (9) that should be investigated in further work.

CLDT is an indisputable reality that renders RP kinetics complicated, but we are adamant that these realities can be conquered, as we have shown here for *E*_a_(<*k*_t_>), hopefully in an esthetically pleasing way.

## Figures and Tables

**Table 1:** Results from low-conversion, solution polymerizations of methyl methacrylate (MMA), *n*-butyl methacrylate (BMA), dodecyl methacrylate (DMA), and styrene (ST), employing *c*_M_ = 0.67 mol L^−1^ in trifluorotoluene (TFT) for MMA, BMA, and DMA, and in EBz for ST; and *c*_I_ = 0.05 mol L^−1^ for AIBN or 0.018 mol L^−1^ for BTMHP, as indicated. Values of *DP*_n_ are those calculated using Equation (7) with the parameter values as per the text.

Monomer (Initiator)	Temperature/°C	*k*_o_/s^−1^	<*k*_t_>/(L mol^−1^ s^−1^)	*DP*_n_
MMA (AIBN)	50	1.93 × 10^−5^	8.85 × 10^7^	102
	50	1.96 × 10^−5^	8.60 × 10^7^	103
	60	5.07 × 10^−5^	9.64 × 10^7^	59
	60	5.32 × 10^−5^	8.75 × 10^7^	62
	70	9.89 × 10^−5^	1.69 × 10^8^	27
	70	1.30 × 10^−4^	9.81 × 10^7^	36
	71	1.14 × 10^−4^	1.54 × 10^8^	27
	85	3.81 × 10^−4^	1.62 × 10^8^	14
	85	2.03 × 10^−4^	5.70 × 10^8^	8
	85	3.46 × 10^−4^	1.96 × 10^8^	13
	85	2.33 × 10^−4^	4.35 × 10^8^	9
	85	3.00 × 10^−4^	2.61 × 10^8^	11
	85	2.47 × 10^−4^	3.85 × 10^8^	9
	85	2.45 × 10^−4^	3.91 × 10^8^	9
	90	5.23 × 10^−4^	1.98 × 10^8^	11
	90	3.74 × 10^−4^	3.88 × 10^8^	8
MMA (BTMHP)	70	6.86 × 10^−5^	1.84 × 10^8^	36
	70	7.93 × 10^−5^	1.38 × 10^8^	42
	85	1.63 × 10^−4^	4.14 × 10^8^	13
	85	1.58 × 10^−4^	4.44 × 10^8^	13
	90	2.66 × 10^−4^	3.41 × 10^8^	12
BMA (AIBN)	50	2.96 × 10^−5^	5.07 × 10^7^	157
	50	3.57 × 10^−5^	3.52 × 10^7^	188
	50	2.80 × 10^−5^	5.74 × 10^7^	147
	60	6.51 × 10^−5^	8.07 × 10^7^	75
	60	7.97 × 10^−5^	5.39 × 10^7^	92
	70	1.77 × 10^−4^	7.44 × 10^7^	49
	70	1.65 × 10^−4^	8.51 × 10^7^	45
	70	1.70 × 10^−4^	8.01 × 10^7^	47
	70	1.44 × 10^−4^	1.12 × 10^8^	40
	85	4.67 × 10^−4^	1.53 × 10^8^	18
	85	4.20 × 10^−4^	1.89 × 10^8^	16
	87	4.74 × 10^−4^	2.09 × 10^8^	14
	90	5.74 × 10^−4^	2.35 × 10^8^	11
	90	5.59 × 10^−4^	2.45 × 10^8^	11
DMA (AIBN)	50	5.53 × 10^−5^	2.62 × 10^7^	292
	60	1.72 × 10^−4^	1.98 × 10^7^	199
	70	3.86 × 10^−4^	2.55 × 10^7^	107
	85	9.94 × 10^−4^	5.25 × 10^7^	37
	85	1.03 × 10^−3^	4.91 × 10^7^	39
	90	1.07 × 10^−3^	1.03 × 10^8^	21
ST (AIBN)	50	3.26 × 10^−6^	4.11 × 10^8^	26
	50	3.74 × 10^−6^	3.13 × 10^8^	29
	60	1.02 × 10^−5^	3.95 × 10^8^	18
	70	2.24 × 10^−5^	6.8 × 10^8^	9
	85	8.66 ×10^−5^	8.76 × 10^8^	5
	85	9.34× 10^−5^	7.51 × 10^8^	5

**Table 2:** Arrhenius parameters *E*_a_ and *A* used in Equation (6) to calculate the propagation rate coefficients, *k*_p_, initiator decomposition rate coefficients, *k*_d_, and initiator efficiencies, *f*, in this work. Also included are *E*_a_ for the monomeric radical termination rate coefficients, *k*_t_^1,1^, that are used for calculations with Equation (9).

Quantity	Material	*E*_a_/(kJ mol^−1^)	*A*	Reference
*k*_p_	MMA	22.36	2.673 × 10^6^ L mol^−1^ s^−1^	[30]
*k*_p_	ST	32.51	4.266 × 10^7^ L mol^−1^ s^−1^	[31]
*k*_p_	BMA	22.9	3.802 × 10^6^ L mol^−1^ s^−1^	[32]
*k*_p_	DMA	21.00	2.512 × 10^6^ L mol^−1^ s^−1^	[32]
*k*_d_	AIBN	130.23	2.89 × 10^15^ s^−1^	[33]
*f*	AIBN	5.70	5.04	[34]
*k*_d_	BTMHP	128.34	2.84 × 10^15^ s^−1^	[33]
*f*	BTMHP	0	0.53	[35]
*k*_t_^1,1^	MMA	9	–	[24]
*k*_t_^1,1^	ST	9	–	[26]
*k*_t_^1,1^	BMA	10	–	[14]
*k*_t_^1,1^	DMA	20	–	[36,37]

**Table 3:** Arrhenius parameters *E*_a_ and *A* for small-chain <*k*_t_>, as derived from Figure 6, together with the corresponding values of the CLDT parameter *e*, as obtained using Equation (10) with parameter values from Table 2.

Monomer	*E*_a_(<*k*_t_>)/(kJ mol^−1^)	*A*(<*k*_t_>)/(L mol^−1^ s^−1^)	*e*
MMA	38 ± 12	8.3 × 10^13^	0.45
BMA	39 ± 7	8.1 × 10^13^	0.45
DMA	32 ± 12	2.3 × 10^12^	0.19
ST	25 ± 6	2.3 × 10^12^	0.34

**Table 4:** Activation energy *E*_a_ for the peak degree of polymerization, *DP*_p_, as derived from Figure 8, together with the resulting values of the CLDT parameter *e*, as obtained using Equation (13) with *E*_a_(*k*_t_^1,1^) from Table 2 and *E*_a_(<*k*_t_>) from Table 3.

Monomer	*E*_a_(*DP*_p_)/(kJ mol^−1^)	*E*
MMA	−52 ± 6	0.56
BMA	−57 ± 1	0.51
DMA	−54 ± 4	0.22
ST	−55 ± 2	0.29

## Data Availability

The original contributions presented in the study are included in the article and Supplementary Materials; further inquiries can be directed to the corresponding author.

## Supplementary Materials

The following supporting information can be downloaded at https://www.mdpi.com/article/10.3390/polym16223225/s1: Table S1: Mark-Houwink parameters *K* and *α* employed for universal calibration in size exclusion chromatography; Figure S1: Arrhenius plot for variation of experimental values of overall termination rate coefficient, <*k*_t_>, with temperature, *T*, for bulk, low-conversion polymerization of MMA; Figure S2: Arrhenius plot for variation of experimental values of overall termination rate coefficient, <*k*_t_>, with temperature, *T*, for bulk, low-conversion polymerization of ST; Figure S3: ESI-MS spectrum of polystyrene (PST) obtained from radical polymerization in EBz at 90 °C with c_AIBN_ = 0.05 mol L^−1^ and c_ST_ = 0.67 mol L^−1^. References [70,71,72,73,74,75,76,77,78,79] are cited in the supplementary materials.
