# Supplementary material for: On the Activation Energy of Termination in Radical Polymerization, as Studied at Low Conversion
Source: Polymers (Basel). 2024 Nov 20;16(22):3225. doi: 10.3390/polym16223225 (PMC11598545; doi:10.3390/polym16223225)
Supplement: Supplementary file 1 [file polymers-16-03225-s001.zip › polymers-3299596-supplementary.pdf]

polymers

MDPI

Supporting Information

On the Activation Energy of Termination in Radical Polymerization, as Studied at Low Conversion

Majed M. Alghamdi [1,2] and Gregory T. Russell [2,*]

[1] Department of Chemistry, Faculty of Science, King Khalid University, P.O.Box 9004, Abha 61413, Saudi Arabia
[2] School of Physical and Chemical Sciences, University of Canterbury, Private Bag 4800, Christchurch 8140, New Zealand
* Correspondence: greg.russell@canterbury.ac.nz

2. Materials and Methods

2.3. Molar Mass Measurement

Electrospray ionization mass spectrometry (ESI-MS) was conducted using a regularly calibrated time-of-flight (TOF) detector in positive ion mode with a *m*/*z* range of 800–4000 Da. The instrument was set with a capillary temperature of 200 °C, a nitrogen flow rate of 4 L min^{-1}, and a capillary voltage of 4500 V. Polymer samples were dissolved in a 1:2 (v/v) mixture of dichloromethane and methanol at a concentration of approximately 300 µg mL^{-1}, with 10 µL of 0.1 mol L^{-1} sodium acetate added. Samples were introduced into the electrospray interface via a syringe pump at a flow rate of 240–300 µL h^{-1}.

Table S1. Mark-Houwink parameters K and α employed for universal calibration in size exclusion chromatography.

Polymer	K / (dL g^{-1})	α	Reference
PST	11.4 × 10^{-5}	0.716	[1]
PMMA	9.44 × 10^{-5}	0.719	[2]
PBMA	14.8 × 10^{-5}	0.664	[1]
PDMA	5.18 × 10^{-5}	0.720	[1]

Copyright: © 2024 by the authors. Licensee MDPI, Basel, Switzerland. This article is an open access article distributed under the terms and conditions of the Creative Commons Attribution (CC BY) license (https://creativecommons.org/licenses/by/4.0/).

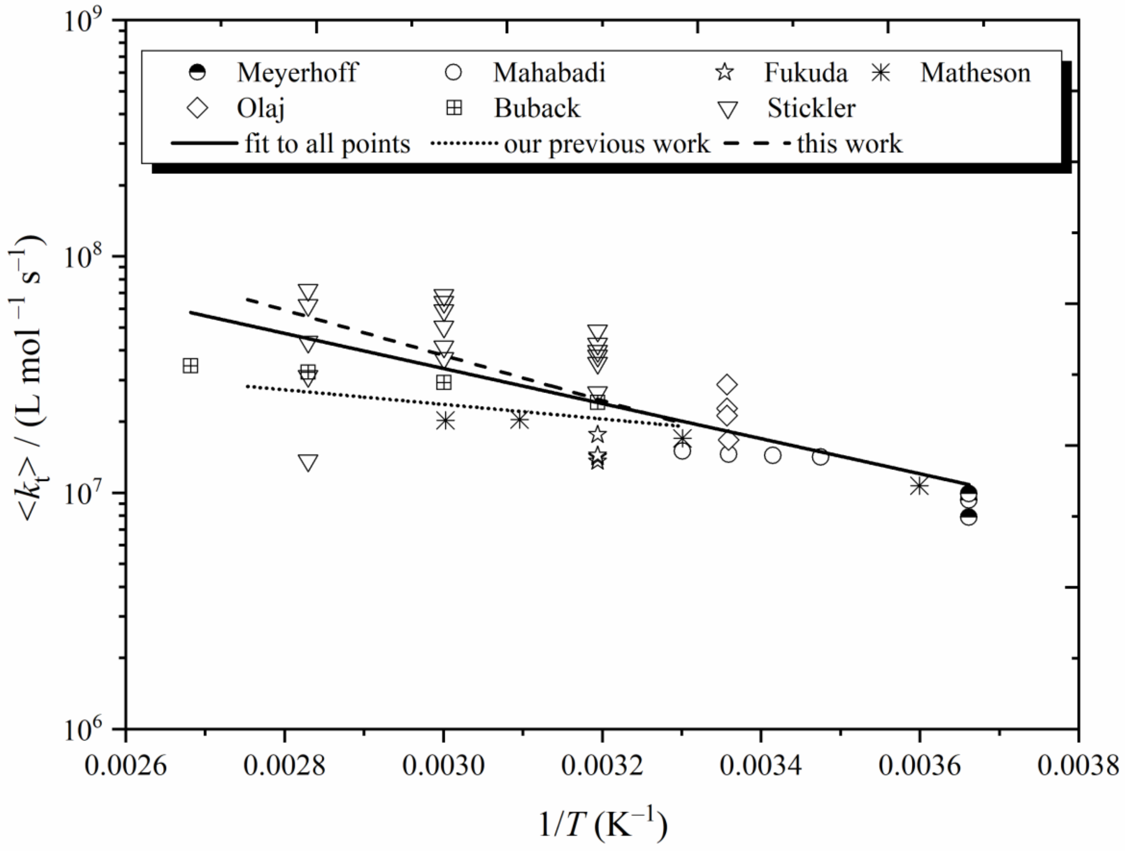

Figure S1. Arrhenius plot for variation of experimental values of overall termination rate coefficient, $<k_t>$, with temperature, T, for bulk, low-conversion polymerization of MMA. Points: values, as indicated, from (left to right, top to bottom) Meyerhoff and Sack-Koulombris [3], Mahabadi and O'Driscoll [4], Fukuda et al. [5], Matheson et al. [6], Olaj and Vana [7], Buback and Kowollik [8], and Stickler [9]. Lines: best fits of all the points (unbroken), of our previous values [10] (dotted), and of the previous values re-analysed in this work (dashed). Note that the latter two lines are just those of Figure 4A.

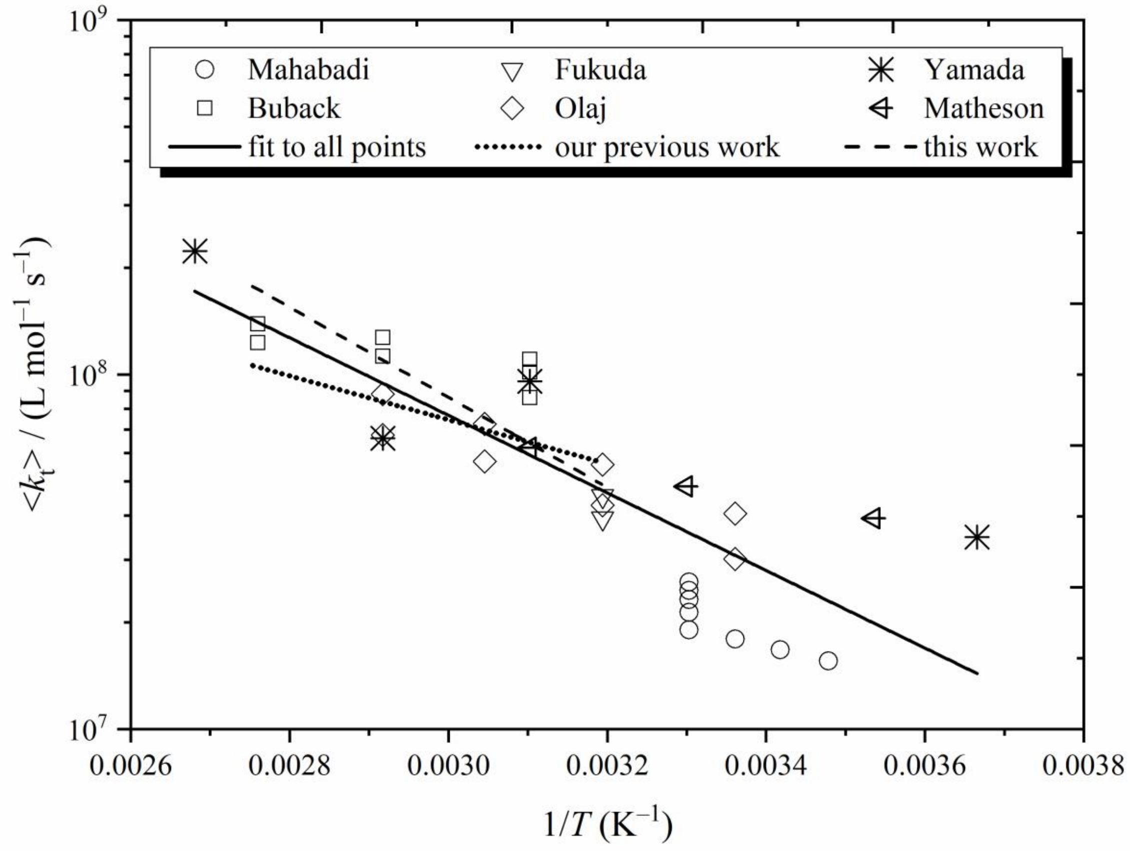

Figure S2. Arrhenius plot for variation of experimental values of overall termination rate coefficient, $<k_t>$, with temperature, *T*, for bulk, low-conversion polymerization of ST. Points: values, as indicated, from (left to right, top to bottom) Mahabadi and O'Driscoll [4], Fukuda et al. [5], Yamada et al. [11], Buback and Kuchta [12], Olaj and Vana [13], and Matheson et al. [14]. Lines: best fits of all the points (unbroken), of our previous values [10] (dotted), and of the previous values re-analysed in this work (dashed). Note that the latter two lines are just those of Figure 4B.

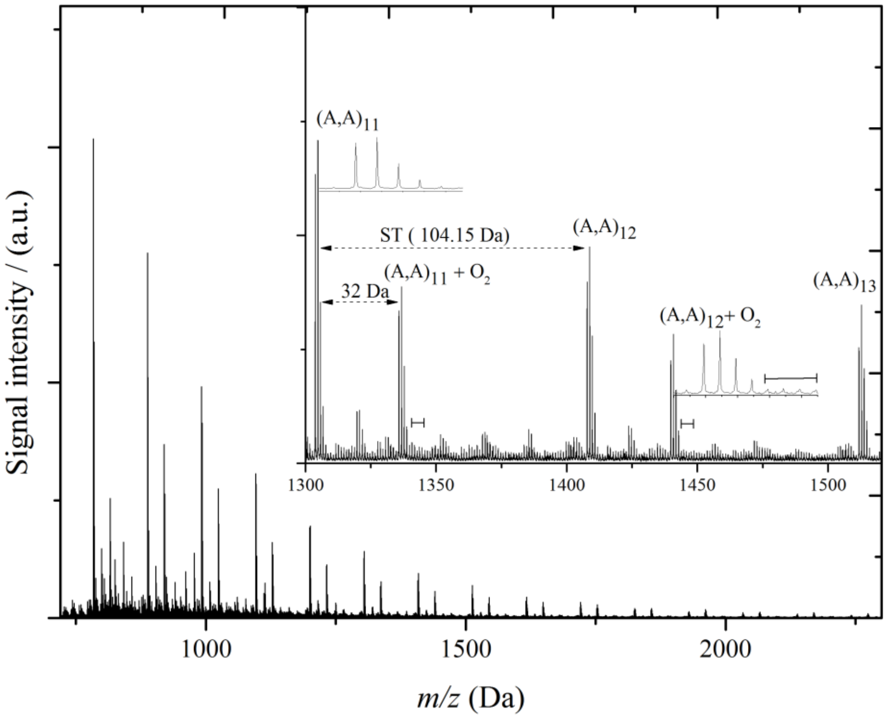

Figure S3. ESI-MS spectrum of polystyrene (PST) obtained from radical polymerization in EBz at 90 °C with c_{AIBN} = 0.05 mol L^{-1} and c_{ST} = 0.67 mol L^{-1}. The close-up shows a portion of the MS spectrum that is typical for two repeat units, where A denotes the primary radical from AIBN, viz. cyanoisopropyl. Thus $(A,A)_{11}$, for example, denotes a PST chain with 11 ST units and A at each end (together with Na^+ from the ESI process). Signals from PST additionally containing O_2 are indicated. Most likely these arise from a propagating radical adding to O_2 and then terminating by combination with another propagating radical.

References

1. Hutchinson, R. A.; Beuermann, S.; Paquet, D. A., Jr.; McMinn, J. H. Determination of Free-Radical Propagation Rate Coefficients for Alkyl Methacrylates by Pulsed-Laser Polymerization. *Macromolecules* **1997,** 30, 3490–3493.
2. Hutchinson, R. A.; McMinn, J. H.; Paquet, D. A., Jr.; Beuermann, S.; Jackson, C. A Pulsed-Laser Study of Penultimate Copolymerization Propagation Kinetics for Methyl Methacrylate/*n*-Butyl Acrylate. *Ind. Eng. Chem. Res.* **1997,** 36, 1103-1113.
3. Sack-Kouloumbris, R.; Meyerhoff, G. Radikalpolymerisation von methylmethacrylat im gesamten umsatzbereich. Stationäre und instationäre experimente zur bestimmung von wachstums- und abbruchsgeschwindigkeit. *Makromol. Chem.* **1989,** 190, 1133-1152.
4. Mahabadi, H. K.; O'Driscoll, K. F. Absolute Rate Constants in Free-Radical Polymerization. III. Determination of Propagation and Termination Rate Constants for Styrene and Methyl Methacrylate. *Journal of Macromolecular Science: Part A - Chemistry* **1977,** 11, 967-976.
5. Fukuda, T.; Ma, Y. D.; Inagaki, H. Free-radical copolymerization. 3. Determination of rate constants of propagation and termination for styrene/methyl methacrylate system. A critical test of terminal-model kinetics. *Macromolecules* **1985,** 18, 17-26.
6. Matheson, M. S.; Auer, E. E.; Bevilacqua, E. B.; Hart, E. J. Rate Constants in Free Radical Polymerizations. I. Methyl Methacrylate. *Journal of the American Chemical Society* **1949,** 71, 497-504.
7. Olaj, O. F.; Vana, P. Chain-length dependent termination in pulsed-laser polymerization, 6: The evaluation of the rate coefficient of bimolecular termination kt for the reference system methyl methacrylate in bulk at 25°C. *Macromol. Rapid Commun.* **1998,** 19, 533-538.
8. Buback, M.; Kowollik, C. Termination Kinetics of Methyl Methacrylate Free-Radical Polymerization Studied by Time-Resolved Pulsed Laser Experiments. *Macromolecules* **1998,** 31, 3211-3215.
9. Stickler, M. Kinetics of radical polymerization of methyl methacrylate initiated with dimethyl 2,2′-azodiisobutyrate. *Makromol. Chem.* **1986,** 187, 1765-1775.
10. Taylor, D. R.; Van Berkel, K. Y.; Alghamdi, M. M.; Russell, G. T. Termination rate coefficients for radical homopolymerization of methyl methacrylate and styrene at low conversion. *Macromol. Chem. Phys.* **2010,** 211, 563-579.
11. Yamada, B.; Kageoka, M.; Otsu, T. ESR study of the radical polymerization of styrene. *Polym. Bull.* **1992,** 29, 385-392.
12. Buback, M.; Kuchta, F.-D. Termination kinetics of free-radical polymerization of styrene over an extended temperature and pressure range. *Macromol. Chem. Phys.* **1997,** 198, 1455-1480.
13. Olaj, O. F.; Vana, P. Chain-length dependent termination in pulsed-laser polymerization, 5a: The evaluation of the rate coefficient of bimolecular termination kt for the reference system styrene in bulk at 25°C. *Macromol. Rapid Commun.* **1998,** 19, 433-439.
14. Matheson, M. S.; Auer, E. E.; Bevilacqua, E. B.; Hart, E. J. Rate Constants in Free Radical Polymerization. III. Styrene1. *Journal of the American Chemical Society* **1951,** 73, 1700-1706.

Disclaimer/Publisher's Note: The statements, opinions and data contained in all publications are solely those of the individual author(s) and contributor(s) and not of MDPI and/or the editor(s). MDPI and/or the editor(s) disclaim responsibility for any injury to people or property resulting from any ideas, methods, instructions or products referred to in the content.
